# Risk Factor-Driven Prehabilitation Prior to Abdominal Wall Reconstruction to Improve Postoperative Outcome. A Narrative Review

**DOI:** 10.3389/jaws.2022.10722

**Published:** 2022-09-16

**Authors:** Allard S. Timmer, Jeroen J. M. Claessen, Marja A. Boermeester

**Affiliations:** ^1^ Department of Surgery, Amsterdam UMC Location University of Amsterdam, Amsterdam, Netherlands; ^2^ Amsterdam Gastroenterology, Endocrinology and Metabolism, Amsterdam, Netherlands; ^3^ Amsterdam Institute for Infection and Immunity, Amsterdam, Netherlands

**Keywords:** review, risk factors, hernia repair, prehabilitation, abdominal wall reconstruction

## Abstract

All abdominal wall reconstructions find themselves on a scale, varying between simple to highly complex procedures. The level of complexity depends on many factors that are divided into patient comorbidities, hernia characteristics, and wound characteristics. Preoperative identification of modifiable risk factors provides the opportunity for patient optimization. Because this so called prehabilitation greatly improves postoperative outcome, reconstructive surgery should not be scheduled before all modifiable risk factors are optimized to a point where no further improvement can be expected. In this review, we discuss the importance of preoperative risk factor recognition, identify modifiable risk factors, and utilize options for patient prehabilitation, all aiming to improve postoperative outcome and therewith long-term success of the reconstruction.

## Introduction

The aim of abdominal wall reconstruction (AWR) is to restore the original anatomy of the abdominal wall. The level of complexity of these procedures varies between simple to highly complex, being affected by a multitude of different factors. These factors are divided into three categories: patient characteristics (comorbidities), hernia characteristics, and wound characteristics. Several patient comorbidities such as smoking, diabetes, and obesity are independent risk factors for the development of postoperative wound complications and increase the risk of hernia recurrence [1–3]. Preoperative recognition of these so-called modifiable risk factors provides an opportunity for patient optimizing and therewith improve postoperative outcome. Most hernia-related characteristics are directly related to previously performed surgeries and cannot be changed. With the introduction of botulinum toxin and tissue expanders, important hernia dimensions as hernia diameter and loss of domain (LOD) have become partly modifiable. The presence of bacterial contamination to the hernia site is the most important wound-related risk factor for postoperative complications [4]. Reasons for contamination are open wounds, presence or creation of stomata, perioperative opening of the gastrointestinal tract, enterocutaneous- or enteroatmospheric fistulas, and infected mesh. Although some of these variables are a fact and cannot be changed preoperatively, others can partly be managed, for example by reducing the bacterial load of infected wounds or infected mesh using negative pressure wound therapy with fluid instillation. Because many different factors add to the level of complexity and therewith the risk of postoperative complications, use of standardized hernia classification systems can help identifying all risk factors that require preoperative modification. However, available hernia classification systems are limited in their use as prehabilitation tool.

The emphasis in reducing the risk of postoperative complications has largely been on specific surgical techniques and choice of mesh prosthetic. Recently, the importance of preoperative risk factor recognition and adequate prehabilitation has gained more and more attention [1, 3, 5]. In this narrative review, we describe the importance of preoperative patient assessment with recognition of modifiable risk factors and discuss future perspectives in AWR, all attempting to minimize the risk of postoperative complications and therewith improve chances of long-term success.

## Hernia Classification Systems

Abdominal wall hernias can present in many different forms with respect to type (primary or incisional), location, and dimensions. Over the years several classification systems have been proposed, attempting to stratify the different types of abdominal wall hernias. The first that acquired general acceptance is the European Hernia Society (EHS) classification [6]. This classification was developed in 2009 by a group of international experts that aimed to improve the possibility of comparing ventral hernia patients from different studies. In this classification, primary hernias are separated from incisional hernias because of differences in etiopathology. Primary ventral hernias are specified according to location (midline or lateral) and diameter (transverse width), whereas incisional hernias are classified according to location, dimension (transverse width and craniocaudal height), and type (first incisional or recurrent). Although the EHS classification is the first to classify ventral hernia according to location, size, and type, it does no incorporate patient specific risk factors, which are now known to have an enormous impact on clinical outcomes after surgical repair.

In 2010, the Ventral Hernia Working Group (VHWG) developed a classification system, attempting to preoperatively assess the risk of postoperative surgical site occurrences (SSO) following open ventral hernia repair. SSO include surgical site infection (SSI), seroma/hematoma, wound dehiscene and formation of an enterocutaneous fistula. Based on specific patient comorbidities and wound characteristics, they proposed a four graded system [7]. Two years later, Kanters et al prospectively assigned 299 patients undergoing open ventral hernia repair to this system, finding that modifying the VHWG system into a three graded system significantly improves the accuracy of predicting the risk of SSO. In the modified classification, grade 1 are patients who lack comorbidities and have a clean hernia site. Patients with one or more specific comorbidities are assigned to grade 2. Patients with a contaminated hernia site are assigned to grade 3. Grade 3 patients are further stratified (a, b, or c) based on the level of contamination. Corresponding risks of SSO after open ventral hernia repair are 14%, 27% and 46%, for grades 1, 2 and 3, respectively (Table 1).

**TABLE 1:** Modified ventral hernia working group grading system, adapted from Kanters et al. [2].

Grade 1: Low risk	Grade 2: Comorbidities	Grade 3: Contamination
SSO: 14%	SSO: 27%	SSO: 46%
No history of wound infection	Smoking	A Clean-contaminated
	Obesity	B Contaminated
	Diabetes mellitus	C Active infection
	COPD	
	History of wound infection	

Abbreviations: SSO, surgical site occurrence; COPD, chronic obstructive pulmonary disease.

Still, the modified VHWG system does not include hernia dimensions or information about previous hernia surgery. Particularly interesting is the Hernia-Patient–Wound (HPW) classification described by Petro et al [8]. The HPW classification is a practical system incorporating both patient comorbidities as well as hernia width and presence of contamination in predicting the risk of wound complications and hernia recurrence (Table 2). In this classification, a stepwise risk increase is seen at cut-off values for hernia width of <10 cm, 10–20 cm and >20 cm. These values are easy to remember and therefore practical in use. However, to date the HPW system lacks external validation.

**TABLE 2:** Hernia-Patient–Wound classification system, adapted from Petro et al. [8].

Stage	Hernia	Patient	Wound	HPW-combinations	SSE rate (%)	Recurrence rate (%)
1	H1	P0	0	H1, P0, W0	5.8	4.7
2	H1 or H2	any P	0	H1, P1, W0 H2, any P, W0	12.6	9.2
3	any H	any P	0 or 1	H1, any P, W1 H2, any P, W1 H3, P0, W0	20.2	13.2
4	H3	any P	0 or 1	H3, P1, W0 H3, any P, W1	38.9	31.1

Transverse hernia width; H1: <10 cm, H2: 10–20 cm, H3: >20 cm. Comorbidities; P0: absent, P1 present. Contamination; W0: absent, W1: present.

Abbreviations: SSE, surgical site events (including all surgical site infections and clinically relevant SSO).

The above-mentioned classification systems can help identify high-risk patients and some offer the possibility to identify and intervene on modifiable risk factors. Furthermore, several risk-assessment models have been developed that aim to identify risk factors but also guide options for prehabilitation. For example, the Ventral hernia risk score is a patient data derived risk assessment tool for wound complications after open hernia repair [9]. Based on several preoperative and operative characteristics, patients are stratified into 5-risk groups for SSI. The Hernia Wound Risk Assessment Tool is another model that builds upon and complements existing risk tools [10]. Derived from data of more than 60.000 patients, the risk of SSO is broken down into 5 categories based on patient characteristics, hernia characteristics, and wound characteristics. Although both tools have good predictive performance, they are not very easy to use. More practical is the Carolinas equation for Determining Associated Risks (CeDAR). This is a mathematical equation, derived from 500 prospectively enrolled patients undergoing open ventral hernia repair, that predicts the risk of postoperative complications and associated costs. The equation is converted to an app that provides simple insight for both surgeons and patients [11]. The CeDAR tool is currently being updated in a larger cohort. Although all these models are evidence-based and validated, there is currently no international consensus on which model should be used. Use of standardized classification systems does not only improve risk assessment and direct preoperative optimization, but also provides a fundamental base for comparing results across different series.

## Preoperative Patient Assessment

Preoperative patient assessment—using the EHS, modified VHWG-, and HPW classifications—focuses on the identification of risk factors for perioperative complications. These risk factors are divided into three categories: patient characteristics, hernia characteristics, and wound characteristics (Table 3). All risk factors that to some extent can be optimized are so-called modifiable risk factors. When present, they should be optimized to a point where no further improvement can be expected before reconstruction is planned. Modifiable risk factors are discussed for each hernia-, patient-, and wound category separately.

**TABLE 3:** Summary of modifiable and fixed risk factors for postoperative wound complications following abdominal wall reconstruction, divided into patient-, hernia-, and wound characteristics.

Modifiable risk factors		
**Patient characteristics**	**Hernia characteristics**	**Wound characteristics**
Smoking	Hernia diameter >10–15 cm	Amount of skin/subcutaneous tissue
Diabetes	Loss of Domain >15–20%	
Obesity		
Cardiopulmonary disease		
Malnutrition		
Drug use		
Anemia, renal insufficiency		
**Fixed risk factors**		
**Patient characteristics**	**Hernia characteristics**	**Wound characteristics**
Age	History of abdominal infection	Stoma(ta) and intestinal fistula(s)
Prior malignancy	Number of previous abdominal surgeries and AWR, atrophy/absence of muscle(s)	Infected mesh
	Previous component separation and mesh use	Quality of skin/subcutaneous tissue

Patient assessment starts with a comprehensive medical history including previously performed abdominal surgeries and reconstructions, and current drug use. Original operation reports with information on mesh type, mesh position and component separation technique (CST) should be requested. Physical examination is performed in both standing and supine position. Photographs should be documented in the patient’s electronic medical record. Attention is paid to the quality and amount of skin and subcutaneous tissue, abdominal wounds, stomata, fistulas, and the location and number of the hernia(s). When the skin is of poor quality and/or wounds are present, major reconstructive surgery may be the only option to close the skin. Depending on these findings, preoperative consultation of a plastic/reconstructive surgeon should be considered.

Besides the identification of risk factors specific to abdominal wall reconstruction, it is important to obtain information on general risk factors for abdominal surgery including hemoglobin level, kidney function, prior malignancy with current status of follow-up. Preoperative colonoscopy should be done if indicated in case of colon cancer follow-up or complaints suspected of concurrent colonic disease before engaging in a hernia repair.

It is essential to obtain cross-sectional imaging of the abdomen [12]. Computed tomography (CT) can determine the exact number, location(s), and dimension(s) of the defect(s). Measurements of transverse hernia width and LOD can help predict whether achieving fascial closure will be likely and direct the need for chemical and/or surgical CST. CT can also identify atrophy or absence of muscles, intestinal fistula(s), previously performed CST, and previously placed mesh(es), all greatly affecting the possibilities for the upcoming reconstruction.

### Patient Characteristics

Prehabilitation options are presented in Table 4.

**TABLE 4:** Preoperative patient assessment, risk factor recognition and options for patient prehabilitation to reduce the risk of postoperative complications following abdominal wall reconstruction.

Preoperative patient assessment	
Comprehensive medical history	Including symptoms, drug use, and previous abdominal operations and AWR (request original surgical reports)
Physical examination	In standing and supine position: number of hernias, size, location, LOD (Valsalva maneuver), wounds, stomas, fistulas, amount and quality of skin
Radiological imaging	Contrast enhanced CT imaging, maximum 6 months old and performed after the last intra-abdominal operation or drainage^a^
**Hernia classification systems**	
EHS, mVHWG, and HPW classification	
**Patient risk factors**	**Prehabilitation options**
Smoking	Cessation at least 4 weeks prior to AWR
	Consider nicotine replacement therapy
Diabetes	Personal assessment by GP or diabetes specialist
	Target HbA1c <7.0% (153 mg/dl)
	HbA1c > 8.0% (185 mg/dl): postpone elective reconstruction
Obesity	BMI 30–40: personalized weight loss interventions
	BMI >40: consider bariatric surgery first
	BMI >50: AWR is advised against; bariatric surgery may be an option
Cardiopulmonary disease	Specialist referral with personalized assessment (CPET) and optimization
Malnutrition	Consider (temporary) enteral or parenteral feeding
	Monitor the effect by weight gain, electrolyte and albumin level
Anticoagulative and/or immunosuppressive drugs	Consider consultation of the prescribing physician to discuss the continuation/discontinuation, or bridging therapy
**Hernia and Wound risk factors**	**Prehabilitation options**
Large fascial defect (>10–15 cm) or large LOD (>15%–20%)	Consider preoperative botulinum toxin^b^, surgical tissue expanders, and/or PPP (only in highly selected cases)
Intestinal fistula(s)	Wound care: consider fistula adaptor/wound manager
	AWR: ≥6–9 months after the last intra-abdominal procedure
	High-output reduction (reduced oral intake, medication (TPN if needed)
Active contamination (e.g., active wound infection, acute mesh infection)	Bioburden reduction as the first step whenever possible (consider the use of V.A.C VeraFlo™)

Abbreviations: LOD, loss of domain; EHS, European hernia society; mVHWG, modified Ventral Hernia Working Grade; HPW, hernia patient wound; GP, general practitioner; CPET, cardiopulmonary exercise testing; PPP, progressive pneumoperitoneum; TPN, total parenteral nutrition.

a Except for small primary midline hernia.

b There is currently no consensus on the indication of botulinum toxin prior to AWR.

#### Smoking

It is well recognized that cigarette smoking is associated with postoperative morbidity such as wound complications and prolonged hospital stay [13, 14]. Specifically for ventral hernia repair, smokers have a significantly higher risk to develop wound complications, cardiopulmonary complications, as well as a recurrent hernia [15, 16]. Given these established relationships, patients need to stop smoking before elective reconstruction is performed. The minimum period of smoking cessation to effectively reduce the risk of SSO is 4 weeks, however, the longer the abstinence, the greater the benefit [3, 17]. There is currently no strong evidence indicating that short-term cessation (<4 weeks) increases or reduces the risk of postoperative complications. Use of nicotine patches does not diminish the effect of cessation, and seems a valid option.

#### Diabetes

Poor glycaemic control in the perioperative period (up to 60 days postoperative) increases the risk of wound complications [5,18,19,20]. Therefore, all patients with known diabetes require personal assessment with referral to a general practitioner or diabetes specialist if results indicate inadequate glucose control. Glycosylated hemoglobin (HbA1c) is a measure that reflects long-term blood sugar levels. Abdominal wall reconstructive experts have agreed that the target HbA1C level is <7.0% (153 mg/dl), and it is recommended not to perform elective repair when HbA1C exceeds 8.0% (185 mg/dl) [3]. A meta-analysis of 15 randomized trials found that intensive perioperative glucose control significantly reduces the risk of SSI in both patients with and without diabetes [21]. It that same study it was found that intensive glucose control is not associated with significantly higher risk of hypoglycemia related serious adverse events.

#### Obesity

Where the incidence of obesity is rapidly growing, so does the importance for well-developed strategies on how and when to perform AWR in this population. Obesity is one of the main risk factors for postoperative SSI, incisional hernia, and hernia recurrence [4, 22, 23]. The higher the BMI, the higher these risks become [24]. A BMI <30 is preferred [3, 23]. Patients with a BMI between 30 and 40 are advised to lose weight before AWR is planned. Each patient may require a different strategy to reach their individual weight goal, and a BMI <30 is not achievable for every patient. A hernia with major LOD also benefits from weight loss but long postponement of surgery has the inherit risk of increased LOD. Lifestyle modification, preferably by consulting a dietician and fitness coach or physiotherapist, is the first step. Losing weight can take a long time, especially when patients are not fully motivated. It is therefore important that patients understand that the risks of postoperative complications are directly associated with a higher BMI. By letting patients set their own target weight they are invited to become part of the solution. A collaboration between actively participating patients and medical specialists has shown to result in successful preoperative and long-term weight loss [25]. When BMI exceeds 40, chances for postoperative complications are so high that elective hernia surgery should not be scheduled. In recent times, particularly for patients with larger hernias (>10–15 cm) and/or large LOD (>15%–20%), a low threshold for referral for bariatric surgery should be considered. In such cases, a straightforward laparoscopic gastric sleeve is advised, instead of a formal gastric bypass. After bariatric surgery has been performed, AWR is scheduled only after significant weight loss is achieved. In patients with a BMI exceeding 50, hernia repair is associated with extremely high perioperative risks and recurrence rates, and as such elective surgery should not be undertaken without extreme weight loss, usually after bariatric surgery first [1, 3]. For all overweight patients, elective AWR should not be scheduled before the target weight is reached or significant weight loss is achieved. As an exception, patients with rapidly progressive LOD or (recurrent) obstruction of bowel loops in the hernia sac may need repair in suboptimal circumstances.

Although bariatric surgery is effective in the treatment of obesity, it is also associated with an unwanted risk of different micronutrient deficiencies as a result of reduced absorption [26]. Also, rapid weight loss without bariatric surgery, associated with reduced oral intake on a low-calorie diet has to some extent the same risk. Therefore, concentrations of vitamins, minerals and spore elements should be closely monitored, and supplemented if indicated, in patients undergoing bariatric surgery and patients with extreme weight loss on low-calorie diet. The most common deficiencies after RYGB and sleeve gastrectomy are vitamin D, followed by vitamin B1 and B6. However, the risk of specific types of nutritional deficiencies depends on which part of the intestine is bypassed.

#### Pulmonary Disease

Chronic obstructive pulmonary disease (COPD) is independently associated with SSI, longer hospital stay, and mortality following hernia repair [27]. Patients with COPD require preoperative consultation by a pulmonologist to determine disease severity (long function tests) and adequate optimization if indicated. Preoperative optimization specifically of COPD is called pulmonary rehabilitation. This is a supervised program of 6–8 weeks and consists of health education, progressive exercise training, and needless to say smoking cessation. It has repeatedly been shown that pulmonary rehabilitation improves clinical outcomes and quality of life [28, 29].

#### Cardiovascular Disease

Impaired cardiac function can have many different causes and may limit performing status from mild to severe. All patients with known or suspected cardiac disease require referral to a cardiac specialist with adequate preoperative testing. Patients with complaints of abdominal angina may have significantly decreased abdominal perfusion and require preoperative arterial phase CT. When occlusion is significant, the possibilities for endovascular treatment prior to surgery should be discussed.

It has been shown that impaired cardiopulmonary condition is associated with poor surgical outcome [30]. A single test to translate physical condition into objective measures is not available yet, as physical condition contains many aspects. For both pulmonary and cardiac disease, cardiopulmonary exercise testing (CPET) is a promising test to measure cardiorespiratory condition and to identify specific causes of dysfunction. It is a dynamic test that measures the pulmonary gas exchange at rest and at increasing levels of exercise. Many variables such as range in heart rate, peak oxygen content and anaerobic threshold can be measured, providing specific information on cardiopulmonary, circulatory, and metabolic systems. Of specific interest is the ventilator anaerobic threshold (VAT), an estimate of the pulmonary exercise capacity. Patients with a low VAT have a higher risk of postoperative complications after major surgery [31, 32]. Furthermore, it has been shown that prehabilitation with preoperative physical exercise programs enhances cardiorespiratory fitness and improves postoperative outcomes [33, 34]. Different studies advocate the preoperative use of CPET, however, interpretation can be complex and the best predictive variable with corresponding threshold varies for different underlying conditions [35,36,37]. Initially, CPET was used to predict the appropriate level of postoperative care (general ward or intensive care unit). Today, it is used to identify undiagnosed or poorly managed cardiopulmonary disease, direct preoperative referrals/interventions, and guide prehabilitation and rehabilitation, and test the efficiency thereof [38].

#### Malnutrition

This is a condition where a person’s uptake of nutrients is insufficient. Because malnutrition cannot be measured using a single parameter, nutritional status is currently assessed on clinical evaluation using weight changes and blood electrolyte levels. Patients with a BMI <18.5 (kg/m^2^), weight loss >10% in the last 6 months, or a serum albumin <30 g/L are at severe nutritional risk and require preoperative nutritional care before surgery is planned [39]. Malnutrition can be seen in patients with significant weight loss in a short time, or after bariatric surgery. In complex hernia patients, malnutrition may results from an insufficient functional bowel length after previous surgery to absorb all necessary nutritional components and fluids. This is termed intestinal failure (IF) and may result from a high-output enterocutaneous- or enteroatmospheric fistula (>500 ml/24 h), a high-output stoma (1500 ml/24 h) or an absolute reduction in (small) bowel length. Patients with these type of fistulas have highly complex hernias and should only be treated in facilities that provide specialized multidisciplinary care regarding (parenteral) nutrition and intestinal fistula surgery [40]. When it is expected that IF patients will not meet their metabolic and nutritional needs by oral intake, temporary intravenous feeding is indicated, with frequent monitoring and supplementation of electrolytes. Enteral feeding is usually not a stable nutritional solution for patients with insufficient functional bowel length. Oral intake according to short bowel principles (an oral fluid restriction of 500–1000 ml, consisting of at least 50% isotonic drinks) can be used in parallel with basic nutritional needs provided by parenteral nutrition. From the results of a systematic review on pharmacotherapy for reduction of high output stomas or intestinal fistulas, de Vries et al. have proposed an algorithm for standard care. It consists of high dose proton pump inhibitors (80 mg per day) combined with a gradually increased dose loperamide (up to 32 mg per day) as a first step, followed by adding codeine (60 mg per day) if output reduction is insufficient [41]. That same systematic review found that several studies reporting on output reduction using somatostatin analogues report inconsistent results and—awaiting more data—it is therefore not recommended in standard care [41]. Increased weight, and stable electrolyte- and albumin status are signs of nutritional optimization.

On the day of surgery, patients are often required to fast an extensive period of time, leading to hunger, thirst, and malaise. Preoperative oral carbohydrate drinks administered up to 2 hours before surgery, are found to be safe and effective in reducing patient discomfort and insulin resistance [42, 43].

#### Drug Use

Performing surgery on patients that use anticoagulative or immunosuppressive medication (including chronic use of steroids, recent use of chemotherapy and antirejection medicines), increases the risk of perioperative bleeding and wound complications, respectively [44, 45]. Specifically for AWR, there are no guidelines on whether these drugs should be continued or temporarily stopped, as their use is based on different indications with individualized risks and benefits. For both type of drugs, it makes sense to discuss the continuation or temporary cessation with the prescribing physician who can also propose the temporary use of an alternative drug and dose (e.g., low molecular weight heparins alternatives for long-acting anticoagulants).

### Hernia Characteristics

**FIGURE 1:**
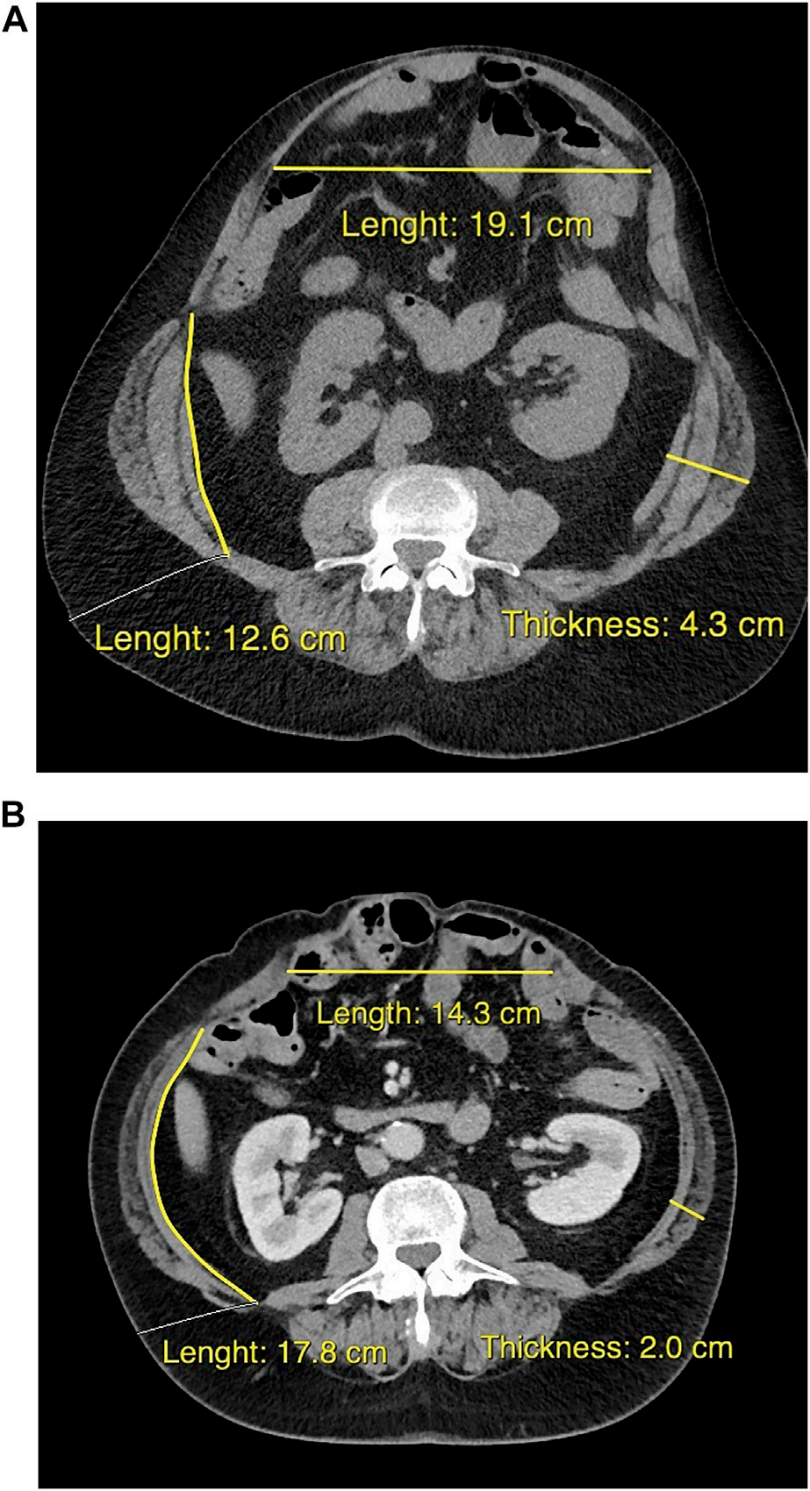
Cross-sectional CT images from the same patient at approximately the same transverse level, when discussed for surgery at the outpatient clinic **(A)**, and about 5 weeks after bilateral intramuscular injection with botulinum toxin **(B)**. The pretreatment flattened and elongated the lateral abdominal wall muscles, and reduced the hernia diameter.

Most hernia-related risk factors are not modifiable. For example, the number of previous hernia repairs with CST and placement of mesh and position with respect to the layers of the abdominal wall are fixed, but significantly affect further surgical possibilities. In order to acquire all necessary information, it is important to obtain the original reports of all previous abdominal surgeries and reconstructions. Furthermore, contrast enhanced CT imaging is essential in the preoperative work-up of complex hernia repair. Transverse hernia width and LOD are CT measures that can help predict whether or not it will be possible to re-approximate the rectus muscles without too much tension on the midline repair [46, 47]. A hernia diameter >10–15 cm and/or LOD >15%–20% may trigger the need to perform a surgical CST, enabling the reduction of the entire hernia volume into the abdominal cavity. Component separation techniques do facilitate fascial medialization, but are associated with an increased risk of wound complications and they should only be used when needed for full fascial closure [48,49,50]. With the introduction of botulinum toxin (BTA) and tissue expanders, these hernia dimension have become partially modifiable. BTA is a protein produced by *clostridium botulinum*. It has a paralyzing effect that is maximal 2–4 weeks after injection which slowly decreases in 3–4 months thereafter. The injections are minimal invasive and can be performed under ultrasonographic guidance allowing dynamic identification of the different muscles. When comparing CT images from before and after BTA treatment, a decrease in lateral abdominal wall muscle thickness and an increase in length is observed [51] (Figure 1). So far, no serious adverse events of these injections prior to AWR have been described in the literature [51]. Weakness of coughing and sneezing are reported, possibly because of impairment of accessorial respiratory muscle function, which suggests cautious use of BTA pretreatment in patients with pulmonary dysfunction or neurodegenerative disease. How the preoperative chemical relaxation of the abdominal wall musculature reflects on hernia recurrence rates and postoperative complications is yet unknown. In patients with extremely large LOD, fascial closure may become near impossible, even with the use of BTA or surgical CST. An option is to combine the use of BTA with progressive pneumoperitoneum (PPP), in which air is repeatedly insufflated into the abdomen. Hereby, the abdominal volume is enlarged providing the possibility for extremely large hernias to be reduced back into the abdominal cavity. PPP is associated with the risk of severe complications and should only be performed in highly selective cases and in experienced centers [52]. Another method for abdominal wall or skin expansion is the use of tissue expanders (TE). These devices are surgically implanted and insufflated in the weeks thereafter. Intramuscular position is mostly used for enlargement of the fascia and muscles, and subcutaneous position for shortage of healthy skin. Although some studies indicate that TE are safe to use, data are limited and its role prior to hernia repair requires further investigation [53, 54].

### Wound Characteristics

Bacterial contamination of the hernia site increases the risk of wound complications, where higher level of contamination correlates with a higher risk [2, 8]. Open wounds, stomata, (inadvertent) opening of the gastrointestinal tract during surgery, intestinal fistulas, and infected mesh are the most common causes of contamination. Wound managers and fistula adaptors can be used to protect and optimize the quality of the skin surrounding a fistula. Although skin closure should be sought for during AWR, this might become difficult in the presence of large skin and/or subcutaneous defects. In challenging cases of skin defects, a plastic surgeon should be consulted preoperatively. Elective reconstructive surgery should not be performed on patients with an active abdominal infection, for instance due to a chronic abdominal wound or a SSI from a recent surgery. Efforts should be made to clear the infection or at least reduce the bacterial load to a minimum before surgery is planned. This can be done by surgical debridement, repeated wound cleansing and/or the use of negative pressure wound therapy with fluid instillation (V.A.C. VeraFlo™).

Infected mesh is a serious complication and its treatment is complex. Different studies have shown the use of negative pressure wound therapy (NPWT) supported by long-term antibiotics to be feasible for mesh salvage. The observational study by Berrevoet et al. reports on a consecutive cohort of 724 patients with a ventral or incisional hernia for which the primary treatment for wound infection was NPWT [55]. Sixty-three patients developed an infectious complication, of which 37 patients had an acute mesh infection. Interesting is that all monofilament large pore polypropylene meshes, of which the majority were positioned in retromuscular position, could be salvaged with NPWT. The only three patients that required removal of the mesh were patients with a polyester-based mesh. Another observational study by Nobaek et al. investigates the use of NPWT for infected synthetic mesh [56]. Their study includes different types of synthetic mesh, including polypropylene, composite mesh, and polyvinylidene difluoride, and finds that 44 of 48 meshes (91%) could be preserved with NPWT. These data indicate that conservative treatment of mesh infection can be successful for some specific types of mesh. Less optimistic is the algorithm proposed by Kao et al. [57] Based on clinical experience of more than 200 patients with a mesh infections—of which 84% eventually needed explantation—they recommend that all infected meshes other than lightweight (large pore) polypropylene should be explanted [57]. Furthermore, in patients who have an intestinal fistula or who smoke, the mesh should be excised regardless of the specific type. Other than this experienced-based algorithm, there are no guidelines specifically on treatment of patients with infected mesh.

## Timing of Hernia Repair

Abdominal wall reconstruction should be performed in elective setting, and only when all modifiable risk factors are optimized. Optimization can take a long time, for example in patients with morbid obesity or malnutrition from intestinal fistulas. Therefore, patients need to be informed about the serious risk of postoperative complications when optimization is unsuccessful so that they become motivated to actively participate in the process. Emergent hernia repair is associated with higher risk of readmission, reoperation, and mortality [58]. The only reason for emergent repair is when patients present with unresolved obstruction or signs of bowel strangulation or ischemia. Infected mesh is not a reason for emergent hernia repair without the necessary prehabilitation.

For patients with intestinal fistulas and/or patients with a large ventral hernia after an open abdomen, it is advised not to perform reconstructive surgery within 6–9 months after the last laparotomy or intra-abdominal drainage. Scar tissue or split skin graft tissue overlying the former open abdomen, covering the viscera, should be softened, and easily lifted from the underlying intestines in-between two fingers (positive lift sign). If this is not the case, the abdominal cavity has not matured yet and is not ready for reconstructive surgery. Patients with acute IF are not fit for surgery. These patients are best treated using the “bridging to surgery” approach in which reconstructive surgery is postponed for many months [59]. The primary focus lies on management of abdominal sepsis and katabolic state, ideally by percutaneous intervention after the emergency surgery period has passed. Secondary, nutritional status is optimized by regain of oral intake, supplemented by parenteral feeding if indicated. Surgery on patients with enterocutaneous fistulas with a longer fistula channel should be delayed for many months because these fistulas may close with conservative management. Furthermore, a longer time period up to reconstruction is associated with lower risk of fistula recurrence [60, 61].

## Promising Predictors for Postoperative Outcome

In search for better ways to predict and improve surgical outcome, frailty and sarcopenia are promising modalities, especially in chronically ill and elderly patients. Frailty is defined as an age-related loss of functional reserve and can predict postoperative outcome [62]. A retrospective study describing 70.339 patients that had undergone a complex hernia repair showed that higher frailty index scores are associated with higher number and higher severity of complications, and mortality [63]. Where the frailty index score is based on 70 different preoperative characteristics, translation into an intervention model is not very convenient. To make the preoperative assessment more practical, the FIT model describes 5 modifiable risk factors: physical condition, nutrition, smoking, anemia and anxiety [64]. These pillars have been tested in a feasibility study including 50 patients scheduled for colorectal surgery. Patients were offered a multimodal prehabilitation program consisting of in-hospital high-intensity endurance and strength training, high-protein nutrition and supplements, smoking cessation in combination with nicotine replacement therapy, screening for anemia and optimization with iron injections if needed, and psychological support. Such prehabilitation program is shown to be feasible, safe, and efficient as 86% of patients recovered to their baseline functional capacity within 4 weeks after surgery, compared to 40% in the control group (*p* < 0.01) [65].

Sarcopenia is the degenerative loss of skeletal muscle mass associated with functional impairment. It can be measured on axial CT images, usually at the level of the third lumbar vertebra, and is associated with poor outcome for several surgical specialties [66–68]. Sarcopenia is not only seen in malnourished patients but also in the obese population where the presence of sarcopenic obesity is a predictor of postoperative morbidity and mortality after bariatric surgery [69]. An association between sarcopenia and postoperative complications following hernia repair has not yet been found, and its role in the preoperative risk assessment in this population remains to be clarified [70].

## Conclusion

The complexity of abdominal wall reconstruction depends on a multitude of variables that can be divided into patient comorbidities, hernia characteristics, and wound characteristics. Many patient comorbidities and some hernia characteristics are modifiable, introducing the possibility for preoperative patient optimization. Use of standardized classification systems can help in the identification of complicating risk factors and guide options for prehabilitation. Because postoperative wound complications may have severe clinical consequences, it is imperative to identify and optimize all modifiable risk factors to a point where no further improvement can be expected before abdominal wall reconstruction is planned.
